# Immunocytochemical staining of proliferating cells in fine needle aspiration smears of primary and metastatic breast tumours.

**DOI:** 10.1038/bjc.1988.115

**Published:** 1988-05

**Authors:** V. Kuenen-Boumeester, D. I. Blonk, T. H. Van der Kwast

**Affiliations:** Department of Cytology, Rotterdam Radio-Therapeutic Institute, The Netherlands.

## Abstract

**Images:**


Br. J. Cancer (1988), 57, 509-511 © The Macmillan Press Ltd., 1988

SHORT COMMUNICATION

### Immunocytochemical staining of proliferating cells in fine needle aspiration smears of primary and metastatic breast tumours

V. Kuenen-Boumeester[1], D.I. Blonk[1] & Th.H. Van der Kwast[2]

[1]*Department of Cytology, Rotterdam Radio-Therapeutic Institute, Box 5201, 3008 AE Rotterdam; and* [2]*Department of Pathology, Erasmus University, Burg. Oudln 50, 3000 DR Rotterdam, The Netherlands*

The variability in biological behaviour of breast carcinomas is a long standing problem. Lymph node status is still the major prognostic indicator (Fisher *et al.*, 1983), but also mitotic activity appears to be of considerable prognostic relevance, either as a single parameter (Schiødt, 1966; Stenquist *et al.*, 1981; Baak *et al.*, 1985), or as part of histological (Bloom & Richardson, 1957; Elston *et al.*, 1982) or cytological (Mauriquand *et al.*, 1986) grading. Since only a minor fraction of proliferating cells is in mitosis, the determination of the total number of proliferating cells could be a better indicator of the growth fraction of a tumour population and therefore might give more information about the biological behaviour of a tumour.

A mouse monoclonal antibody (Ki-67) has become available defining a nuclear antigen present in proliferating cells throughout the cell cycle. The antigen is absent in G0 and early G1 (Gerdes *et al.*, 1983, 1984). Thus, Ki-67 enables the immunocytochemical detection of cycling cells without the need of external administration of radioactively labelled nucleotides or mutagenic substances such as bromodeoxyuridine or iododeoxyuridine. Until now studies on breast tumours using Ki-67 as a proliferation marker were performed on histological material. As fine needle aspiration (FNA) smears are more and more used as diagnostic tools, we investigated the feasibility of the use of this monoclonal antibody on cytological material obtained from benign and malignant tumours as well as from distant metastases of breast carcinomas. In addition, a possible relationship between the Ki-67 determined growth fraction of breast carcinomas and clinical parameters such as tumour size, lymph node status and menopausal status was investigated.

The material consisted of FNA smears of 38 breast carcinomas (31 invasive ductal, 5 colloid and 2 medullary carcinomas), 20 fibroadenomas and 26 metastases of breast carcinomas (16 lymph nodes, 2 liver metastases and 8 local recurrences); the cellularity of aspirates from metastases and primary carcinomas was comparable. The air dried smears were fixed in acetone for 5 min and immunostaining with the mouse monoclonal antibody Ki-67 (Dako, Denmark) diluted 1:10 in PBS, pH 7.4, containing 0.05% gelatin and 0.1% NaN3, was performed using the indirect conjugated immunoperoxidase method as described previously (Van der Kwast *et al.*, 1985). After addition of diaminobenzidine as substrate brown speckled staining of nuclei or nucleoli was considered as a positive reaction with Ki-67. Mitotic figures were also stained. Nuclear counterstaining was achieved by a 1 min incubation in Mayer's haematoxylin (Figure 1). The percentage of Ki-67 positive nuclei was determined by counting 500 cells at a magnification of 1000×. The counting was done by a cytologist (VK) and care was taken to count tumour cells only or in the case of benign tumours to count breast epithelial cells only. The clinical data collected from the patients with the primary tumours are shown in Table I.

Correspondence: V. Kuenen-Boumeester.

Received 8 September 1987; and in revised form 18 January 1988.

**Figure 1** Picture of FNA smear of breast carcinoma, showing Ki-67 stained nuclei (arrows).

**Table I** Clinical data of 38 patients with primary breast carcinomas

| Premeno | Postmeno | T? | T1 | T2 | T3 | T4 | N? | N0 | N1 | N2 |
|---|---|---|---|---|---|---|---|---|---|---|
| 10 | 28 | 2 | 11 | 18 | 3 | 4 | 3 | 14 | 11 | 10 |
| Total | 38 | | | | | 38 | | | | 38 |

T? = Unknown; N? = Unknown.

The FNA smears of the benign tumours contained a low percentage of immunostained nuclei, ranging from 0.0–6.7 with a mean of 1.1 and a median of 0.4. In contrast only four of the malignant tumours showed a percentage below 2, 21 between 2 and 10 and 13 even higher than this (Figure 2) with a mean of 10.5 and a median of 7.9. In comparison with the malignant primary tumours the metastases revealed even higher values with a mean of 14.3 and a median percentage of 12.3. The difference of the last two median values, assessed by the Whitney-test was statistically significant ($P < 0.05$).

Assessment of the Ki-67 determined proliferative tumour cell fraction in FNA smears of malignant breast lesions led to essentially similar results as recently reported in several studies (Gerdes *et al.*, 1986; Lelle *et al.*, 1987; McGurrin *et al.*, 1987; Barnard *et al.*, 1987) of Ki-67 determination on cryostat sections of breast tumours (Table II). It appears from the data in Table II that the Ki-67 percentages of cryostat sections tend to be higher than those of the aspirates. This lower figure in aspirates may be explained by sampling differences. When the tumour is aspirated the material is derived from both peripheral and central parts of the tumour, whereas a cryostat section gives information on one part of the tumour only.

According to Baak *et al.* (1985) and Van der Linden *et al.* (1987) the mitotic activity index in histological material is the strongest independent prognostic factor in their group of patients both retrospectively and prospectively. Although the prognostic significance of the Ki-67 determined proliferative tumour cell fraction remains to be proven, we think that determination of the total cycling fraction of a tumour gives more information about prognosis than assessment of

**Figure 2** The percentages of Ki-67 stained nuclei in benign, malignant and metastatic breast tumours. A = benign ($n = 20$); B = malignant ($n = 38$); and C = metastases ($n = 26$). Arrow signifies median.

**Table II** Reported data of mean percentages of Ki-67 stained nuclei in primary breast carcinomas in cryostat sections and imprints/FNA smears

| | *Histology* | *Cytology* |
|---|---|---|
| Gerdes | 16.6 | |
| Lelle | 16.2 | 14.5[a] |
| McGurrin | 22.0 | |
| Barnard | 20.6 | |
| This study | | 10.5[b] |

[a]Imprints; [b]FNA smears.

numbers of mitotic figures alone. This holds especially true for FNA smears, since they rarely contain mitotic tumour cells. Further support for the hypothesis that the Ki-67 determined proliferative fraction may be of prognostic relevance can be drawn from the *in vitro* $^3$H-thymidine labelling studies of Meyer *et al.* (1983) and Tubiana *et al.* (1984). These authors incubated small specimens of freshly obtained breast tumour tissue with $^3$H-thymidine and counted the number of labelled nuclei from autoradiographed microscopic sections. In the study of Meyer *et al.* (1983) with a follow-up period of 4 years both the lymph node status and the $^3$H-thymidine labelling index appeared to be the strongest independent indicators of early relapse. In the long-term prospective study of Tubiana *et al.* (1984) the $^3$H-thymidine labelling index appeared to be the most predictive independent indicator with respect to relapse-free survival and total survival. It should be noted that Ki-67 immunostaining is only indirect evidence of proliferation while the mitotic index and $^3$H-thymidine incorporation directly relate to proliferative activity. Furthermore, Ki-67 stains cells in G1-phase, cells arrested in this phase of the cell cycle may also be stained by Ki-67 which leads to an overestimation of the fraction of actually proliferating cells.

In contrast to the findings of Lelle *et al.* (1987), but in agreement with the study of McGurrin *et al.* (1987) and Barnard *et al.* (1987), we found no relation between lymph node status and percentage of Ki-67 labelled tumour cells. Neither did the $^3$H-thymidine labelling studies (Meyer *et al.*, 1983; Tubiana *et al.*, 1984) show any relation to lymph node status.

No clear-cut correlation was found in this study between tumour size and menopausal status or percentage of Ki-67 immunostained nuclei. Probably the number of investigated tumours was too low to establish such a relationship. It was interesting to see, however, that the really high values (above 40%, see Figure 3) were only seen in premenopausal women. In addition, the two highest values (30.5% and 19%) in the group of postmenopausal women were medullary carcinomas. It is known that the latter tumours have a high mitotic activity and display a different biological behaviour than the infiltrating duct carcinomas (Azzopardi, 1979).

Our observation of significantly higher percentages of Ki-67 labelled tumour cells in metastases compared to primary carcinomas may point to two possibilities: tumours with a higher proliferative activity may tend to a more aggressive biological behaviour, or the higher proliferative activity of metastasized tumour cells is caused by a more favourable environment. In support of the first possibility is the prospective study of Tubiana *et al.* (1984) which showed that the $^3$H-thymidine labelling index is related to the probability of metastatic dissemination. In addition, Meyer *et al.* (1983) showed that the $^3$H-thymidine labelling index of primary breast tumours and their corresponding axillary metastases were not significantly different. Thus, it seems unlikely that the higher proliferative activity of metastasized tumours can be attributed to microenvironmental influences. Nevertheless,

**Figure 3** Percentage of Ki-67 stained nuclei of the primary carcinomas *versus* menopausal status. M = medullary carcinoma.

it would be interesting to compare the Ki-67 immunostaining results of the aspirates of the primary tumours and the metastases from the same patient.

Further studies are indicated to demonstrate the prognostic relevance of the immunocytochemical assessment of Ki-67 determined proliferative fraction of tumour cells in FNA smears.

We wish to express our gratitude to Miss C. Trappenburg and to A.C.M. van Nispen for their technical assistance.

### References

AZZOPARDI, J.G. (1979). Problems in breast pathology. *Major problems in pathology*, p. 288. W.B. Saunders: Philadelphia.

BAAK, J.P.A., VAN DOP, H., KURVER, P.H.J. & HERMANS, J. (1985). The value of morphometry to classic prognosticators in breast cancer. *Cancer*, **56**, 374.

BARNARD, N.J., HALL, P.A., LEMOINE, N.R. & KADAR, N. (1987). Proliferative index in breast carcinoma determined *in situ* by Ki-67 immunostaining and its relationship to clinical and pathological variables. *J. Pathol.*, **152**, 287.

BLOOM, H.J.G. & RICHARDSON, W.W. (1957). Histological grading and prognosis in breast cancer: A study of 1409 cases of which 359 have been followed for 15 years. *Br. J. Cancer*, **11**, 359.

ELSTON, C.W., GRESHAM, G.A., RAO, G.S. & 4 others (1982). The cancer research campaign (King's/Cambridge) trial for early breast cancer: Clinico-pathological aspects. *Br. J. Cancer*, **45**, 655.

FISHER, B., BAUER, M., WICKERHAM, D.L., REDMOND, C.K., FISHER, E.R. & cooperating investigators. (1983). Relation of number of positive axillary nodes to the prognosis of patients with primary breast cancer. *Cancer*, **52**, 1551.

GERDES, J., SCHWAB, U., LEMKE, H. & STEIN, H. (1983). Production of a mouse monoclonal antibody reactive with a human nuclear antigen associated with cell proliferation. *Int. J. Cancer*, **31**, 13.

GERDES, J., LEMKE, H., BAISCH, H., WACKER, H.H., SCHWAB, U. & STEIN, H. (1984). Cell cycle analysis of a cell proliferation-associated human nuclear antigen defined by the monoclonal antibody Ki-67. *J. Immunol.*, **133**, 1710.

GERDES, J., LELLE, R.J., PICKARTZ, H. & 5 others (1986). Growth fractions in breast cancers determined *in situ* with monoclonal antibody Ki-67. *J. Clin. Pathol.*, **39**, 977.

LELLE, R.J., HEIDENREICH, W., STAUCH, G. & GERDES, J. (1987). The correlation of growth fractions with histologic grading and lymph node status in human mammary carcinoma. *Cancer*, **59**, 83.

McGURRIN, J.F., DORIA, M.I., DAWSON, P.J. & 4 others (1987). Assessment of tumour cell kinetics by immunohistochemistry in carcinoma of breast. *Cancer*, **59**, 1744.

MEYER, J.S., FRIEDMAN, E., McCRATE, M.M. & BAUER, W.C. (1983). Prediction of early course of breast carcinoma by thymidine labelling. *Cancer*, **51**, 1879.

MOURIQUAND, J., GOZLAN-FIOR, M., VILLEMAIN, D. & 4 others (1986). Value of cytoprognostic classification in breast carcinomas. *J. Clin. Pathol.*, **39**, 489.

SCHIØDT, T. (1966). Breast carcinoma. A histologic and prognostic study of 650 followed-up cases. Ejnar Munksgaard, Copenhagen.

STENKVIST, B., BENGTSSON, E., ERIKSSON, O., JARKRANS, T., NORDIN, B. & WESTMAN-NAESER, S. (1981). Correlation between cytometric features and mitotic frequency in human breast carcinoma. *Cytometry*, **1**, 287.

TUBIANA, M., PEJOVIC, M.H., CHAVAUDRA, N., CONTESSO, G. & MALAISE, E.P. (1984). The long term prognostic significance of the thymidine labelling index in breast cancer. *Int. J. Cancer*, **33**, 441.

VAN DER KWAST, TH.H., VAN VLIET, E., CRISTEN, E., VAN EWIJK, W. & VAN DER HEUL, R.O. (1985). An immunohistologic study of the epithelial and lymphoid components of six thymomas. *Human. Pathol.*, **16**, 1001.

VAN DER LINDEN, J.C., BAAK, J.P.A., LINDEMAN, J., HERMANS, J. & MEYER, C.J.L.M. (1987). Prospective evaluation of prognostic value of morphometry in patients with primary breast cancer. *J. Clin. Pathol.*, **40**, 302.


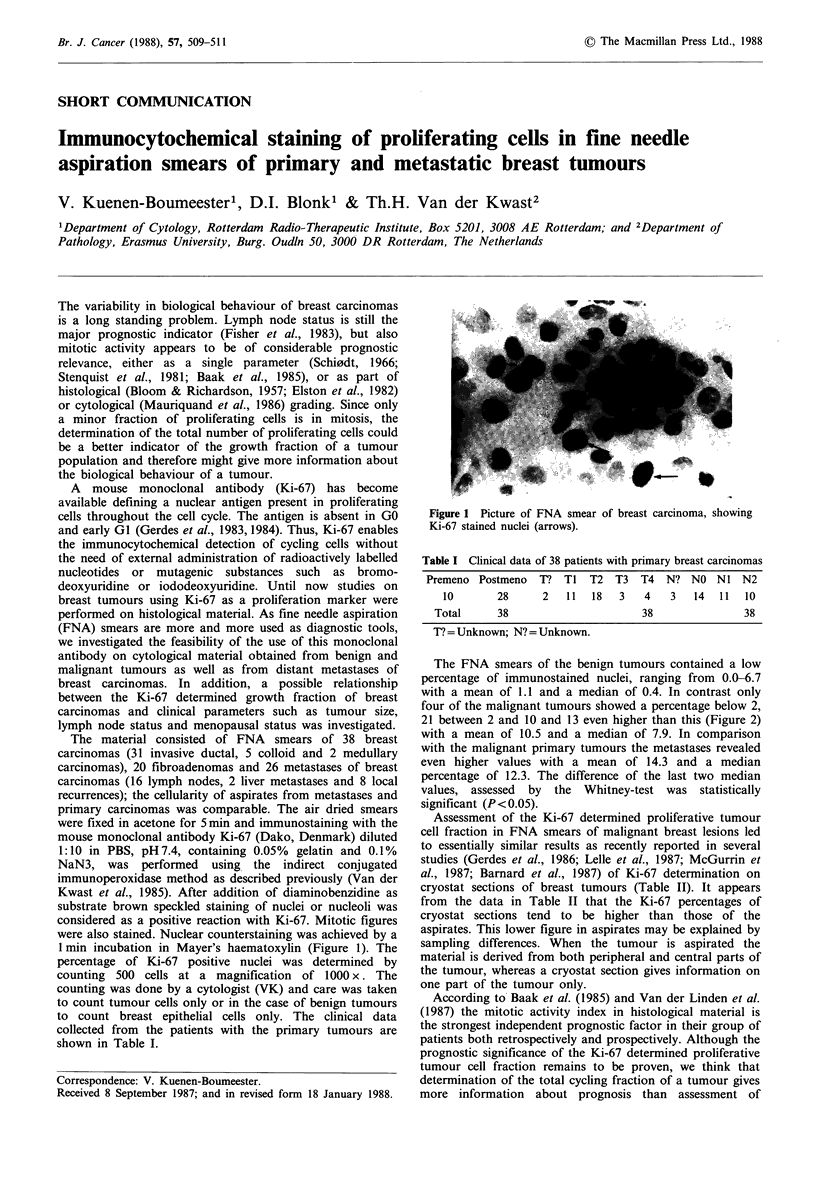

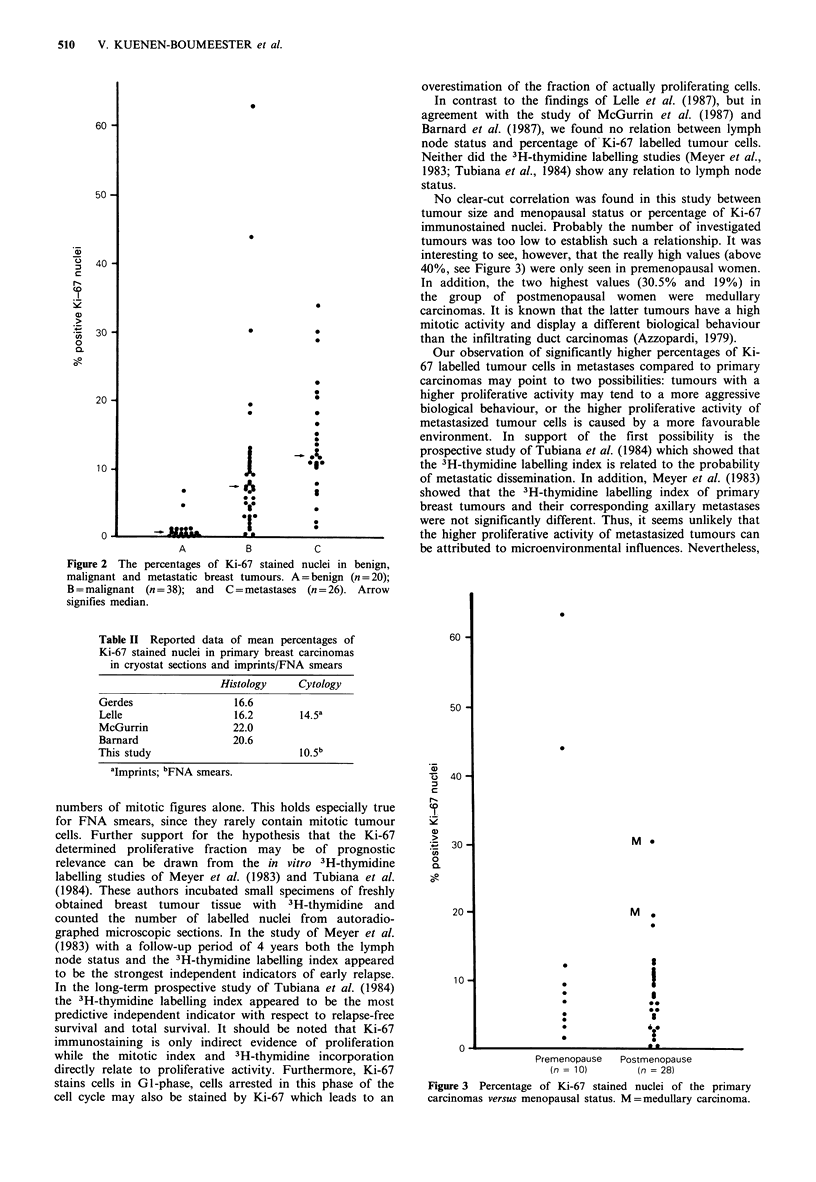

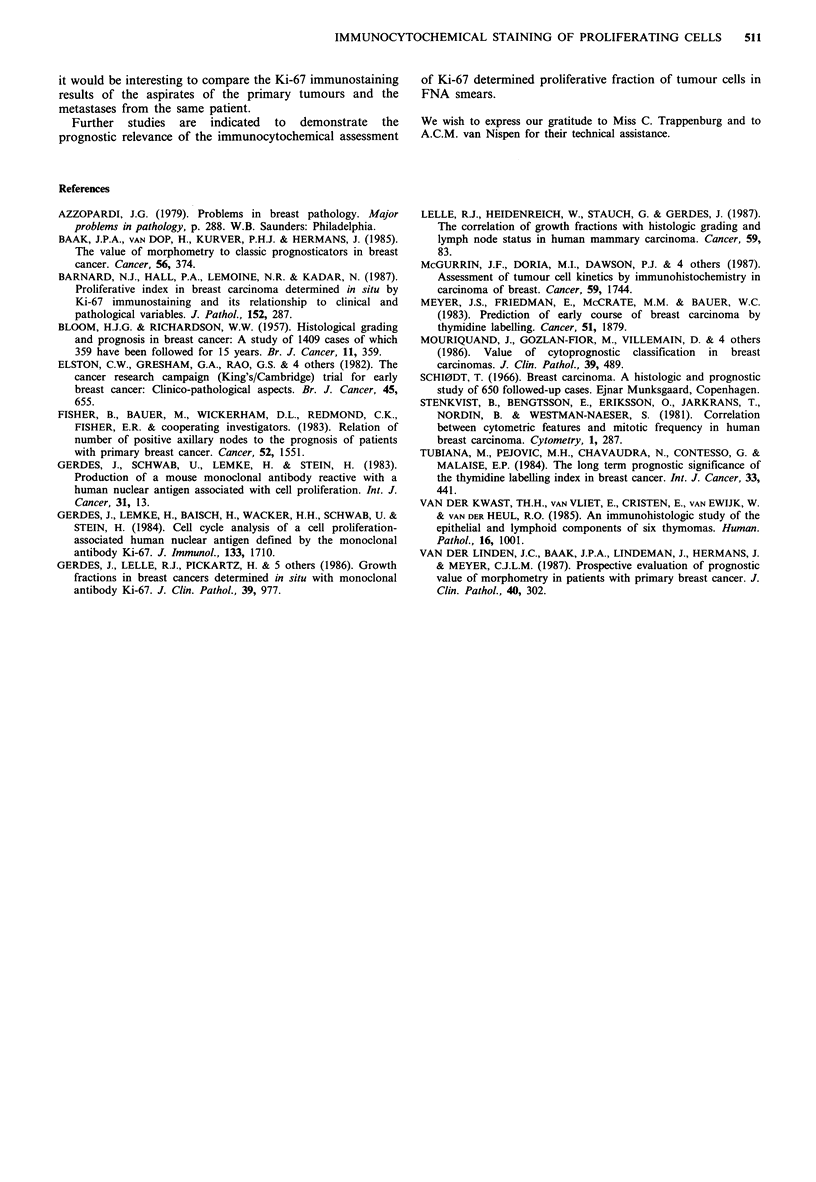


## References

[OCR_00345] BLOOM H. J., RICHARDSON W. W. (1957). Histological grading and prognosis in breast cancer; a study of 1409 cases of which 359 have been followed for 15 years.. Br J Cancer.

[OCR_00334] Baak J. P., Van Dop H., Kurver P. H., Hermans J. (1985). The value of morphometry to classic prognosticators in breast cancer.. Cancer.

[OCR_00339] Barnard N. J., Hall P. A., Lemoine N. R., Kadar N. (1987). Proliferative index in breast carcinoma determined in situ by Ki67 immunostaining and its relationship to clinical and pathological variables.. J Pathol.

[OCR_00350] Elston C. W., Gresham G. A., Rao G. S., Zebro T., Haybittle J. L., Houghton J., Kearney G. (1982). The cancer research campaign (King's/Cambridge trial for early breast cancer: clinico-pathological aspects.. Br J Cancer.

[OCR_00356] Fisher B., Bauer M., Wickerham D. L., Redmond C. K., Fisher E. R., Cruz A. B., Foster R., Gardner B., Lerner H., Margolese R. (1983). Relation of number of positive axillary nodes to the prognosis of patients with primary breast cancer. An NSABP update.. Cancer.

[OCR_00374] Gerdes J., Lelle R. J., Pickartz H., Heidenreich W., Schwarting R., Kurtsiefer L., Stauch G., Stein H. (1986). Growth fractions in breast cancers determined in situ with monoclonal antibody Ki-67.. J Clin Pathol.

[OCR_00368] Gerdes J., Lemke H., Baisch H., Wacker H. H., Schwab U., Stein H. (1984). Cell cycle analysis of a cell proliferation-associated human nuclear antigen defined by the monoclonal antibody Ki-67.. J Immunol.

[OCR_00362] Gerdes J., Schwab U., Lemke H., Stein H. (1983). Production of a mouse monoclonal antibody reactive with a human nuclear antigen associated with cell proliferation.. Int J Cancer.

[OCR_00379] Lellé R. J., Heidenreich W., Stauch G., Gerdes J. (1987). The correlation of growth fractions with histologic grading and lymph node status in human mammary carcinoma.. Cancer.

[OCR_00385] McGurrin J. F., Doria M. I., Dawson P. J., Karrison T., Stein H. O., Franklin W. A. (1987). Assessment of tumor cell kinetics by immunohistochemistry in carcinoma of breast.. Cancer.

[OCR_00390] Meyer J. S., Friedman E., McCrate M. M., Bauer W. C. (1983). Prediction of early course of breast carcinoma by thymidine labeling.. Cancer.

[OCR_00395] Mouriquand J., Gozlan-Fior M., Villemain D., Bouchet Y., Sage J. C., Mermet M. A., Bolla M. (1986). Value of cytoprognostic classification in breast carcinomas.. J Clin Pathol.

[OCR_00403] Stenkvist B., Bengtsson E., Eriksson O., Jarkrans T., Nordin B., Westman-Naeser S. (1981). Correlation between cytometric features and mitotic frequency in human breast carcinoma.. Cytometry.

[OCR_00409] Tubiana M., Pejovic M. H., Chavaudra N., Contesso G., Malaise E. P. (1984). The long-term prognostic significance of the thymidine labelling index in breast cancer.. Int J Cancer.

[OCR_00415] van der Kwast T. H., van Vliet E., Cristen E., van Ewijk W., van der Heul R. O. (1985). An immunohistologic study of the epithelial and lymphoid components of six thymomas.. Hum Pathol.

[OCR_00421] van der Linden J. C., Baak J. P., Lindeman J., Hermans J., Meyer C. J. (1987). Prospective evaluation of prognostic value of morphometry in patients with primary breast cancer.. J Clin Pathol.

